# Observations on the Fevers of Hot Climates

**Published:** 1803-07-01

**Authors:** P. O'Berne

**Affiliations:** late Surgeon of his Majesty's Ship Cerberus


### Observations *on the* Fevers *of* hot Climates; *communicated by* Mr. P. O'Berne, *late Surgeon of his Majesty's Ship Cerberus.*

The more severe in symptoms, and dangerous in effect, any disease is, the more necessary the investigation of and researches after methods of cure must be fully impressive on every mind; it is scarcely necessary to add, that perhaps none comes more strongly under this description than that generally termed Yellow Fever, none therefore more interesting claims your attention.

In the commencement, generally nausea, pain in the head, loins, and hams, succeed; dry surface, increased pulse, but not to be depended on, varying from 80 to 140, chills, anxiety, sighing, prostration of strength; vomiting soon soon takes place, and not unfrequently is the first indication of the disease. The vessels of the tunica conjunctiva become turgid, and a yellow tinge of that membrane takes place, frequently extending over the body. Notwithstanding this circumstance gives rise to the name usually given this complaint, it is by no means a constant attendant, and in many totally wanting. Watchfulness and desire of sleep, without being able to effect it; whilst in others, constant dozing, pain and sensation of heat in the stomach, great thirst; vomited matter gradually changes from yellow to dark green, and at length perfect black. Clammy skin; sometimes petechiæ, but unfrequent; stupor, or violent delirium succeed; paroxysms of vomiting become more rapid, and many expire in one of those paroxysms too shocking to describe, whilst others placidly resign exhausted nature.

No disease perhaps exhibits a greater variety of symptoms, and often less to be depended on than this; sometimes it goes on with every favourable appearance, suddenly changes to the worst, and patients, apparently almost in a state of convalescence, expire in an hour or two. This is a melancholy fact.

The symptoms that we may call favorable are, settled state of the stomach, lessened head-ach, eyes lively, formation of pustules over the surface, or that eruption known in tropical climes by the name Prickly Heat, I have ever remarked as almost a certain indication of recovery; bilious flux, copious and high coloured urine, free perspiration, and sound sleep.

The dangerous, and I am sorry to add, most common symptoms, are severe head-ach, frequent vomiting, heat increasing to a burning sensation, extending down the trachea and alimentary canal; matter vomited and fæces becoming dark, frequent sighing, dull or glassy eye, pale and little urine, dark furr on the tongue, muscular and nervous debility, intermittent pulse, clammy feet, cold sweats, stupor or violent delirium, singultus, coma.

That dark matter vomited, termed Black Vomit, it may be necessary to remark, although laid down by most authors as a certain fatal sign, is by no means so, as I have seen many recover after it; it is also said, "that a diarrhœa almost precludes any hopes of recovery." If by diarrhœa is understood a simple (or bilious) flux, I have ever observed it a decided fortunate event; certainly a flux of putrid dark fæces is extremely bad, and yet even this I have many times seen prove salutary.

 On

On dissection, I have universally found the coats of the œsophagus and alimentary canal corroded; rectum frequently separated from the surrounding muscular substance, and stomach and intestines loaded with black fetid matter. Inflammatory appearance of the membranes of the brain I never saw in the true Yellow Fever, the stomach being evidently the chief seat of the disease.

The most material comes next to our view, and it is painful to say often the most dispiriting; for surely nothing can be more so to a medical man, than to find his utmost exertions in vain, and unremitting attention unsuccessful; it is a melancholy reflection, that the utmost assiduity cannot counterbalance, nor in many instances even mitigate the effects of these baneful climes; however, let even this, instead of damping, stimulate us in the most beneficent of human pursuits, that of endeavouring to alleviate the sufferings of our fellow creatures.

Our first and principal attention should be directed to clearing the first passages, and to keep them free during the disease, being of the greatest importance.

Emetics are by many laid entirely aside on the principle of increasing the already irritable state of the stomach. That a great deal of caution and discrimination in their use is extremely necessary, must be allowed; but I am decididly of opinion much benefit is to be obtained by them. Where nausea or slight vomiting occurs, ipecacuanha is the best; but if the vomiting be more severe, an infusion of chamomile will answer every intention.

Cathartics; calomel combined with pulvis jalapii is perhaps one of the best; the irritating quality of the neutral salts seldom make them advisable.

Blood-letting has been advised by some of the most respectable authorities, I shall therefore only observe, I never saw it used with advantage; on the contrary, always thought it of disservice.

Our next intentions must be directed toward lessening the irritable state of the stomach, supporting the strength, and resisting that tendency to putrescency that exists in this disorder.

Notwithstanding the great variety of opinions that have been, and still are on this subject, calomel will still perhaps be found the most successful medicine hitherto employed, and in general I have but little doubt its want of success in many instances may be attributed to the manner of giving it, or want of attention to the state of the bowels. Calomel, if not given in large quantities quickly repeated, and had better not be given at all; I have used from five to eight grains every two hours, (and sometimes every hour) combined with three grains of the pulvis antimonialis, until a diaphoresis was induced, when the latter was omitted, and the calomel continued until the effect was evident, as metallic taste, fetid breath, or sore mouth. When a gentle salivation is raised, desist in frequency, yet continue so as to keep up the effect of the mercury; the criterion of its success may be determined by its action or non-action. When a speedy and copious salivation comes on, the most happy effects may be looked for; while the contrary prove the reverse. And here again let me observe, that the most minute attention must be paid to keep the bowels free, for which purpose enemas are by far the best.

The kali preparatum, given in a state of effervescence with lemon or lime juice, I have used with great benefit in allaying the heat and lessening the irritable state of the stomach; a solution of magnesia, arabic gum, and mint water, I have found eminently serviceable for the latter purpose.

Blisters, although uncertain, are of great utility both in preventing delirium and lessening vomiting, applied to the region of the stomach.

General warm bath is of the utmost service, or where that cannot be conveniently had, washing all over with warm water.

Of all remedies in use for this disease excepting calomel, perhaps none are of more real service than enemas, and the more simple the better, such as warm water, oil, and vinegar; but on the increased vascular action and heat subsiding, enemas composed of orchis, sago, or portable broth; this last I have found of such uncommon service as makes me wish most strongly to impress the use of it; in many cases where animation seemed nearly exhausted, recovery was the unexpected and welcome effect of this salutary practice.

Opium I have found of little if any service in any stage; this however must be left to circumstances, the acuteness of pain in some instances may require its use, and I never perceived any bad effects caused by it.

Camphor where severe head-ach and depression of spirits occur, I think of much service; and in the latter, vitriolic ether.

Cinchona appears to me evidently of disservice until the patient is in a convalescent state.

Quassia in a cold infusion is a grateful and most useful medicine during convalescence, and here the cold bath is highly serviceable.

The diet from the first should be nourishing and light. Gruel made from the orchis with burnt bread and Madeira, is in general an acceptable beverage; wine should be used with great caution; also all kinds of acids, which when used sparingly are grateful and serviceable; but if used too freely are of real detriment; the utmost precaution must be taken to prevent the patient's strength being too much lowered, to which many fall victims after the fever being totally removed; and among the modes of prevention, I know of none equal to the enemas of portable broth, which will be found most beneficial. I shall conclude by observing, that in a disease like this, whose progress is so rapid, we require to be actively alive to every alteration, and our treatment equally quick.

